# Effect of Fetal Mouse Lung Tissue Co-Culture on *In Vitro* Maturation of Mouse Immature Oocytes

**DOI:** 10.22074/cellj.2017.3866

**Published:** 2017-08-19

**Authors:** Masomeh Belbasi, Seyed Gholam Ali Jorsaraei, Maryam Gholamitabar tabari, Ramzan Khanbabaei

**Affiliations:** 1 Department of Biology, Qaemshahr Branch, Islamic Azad University, Qaemshahr, Iran; 2 Infertility and Reproductive Health Research Center, Health Research Institute, Babol University of Medical Science, Babol, Iran

**Keywords:** In Vitro Maturation, Lung Tissue, Co-Culture

## Abstract

**Objective:**

The aim of this study was to evaluate the fetal mouse lung tissue co-culture on *in vitro* maturation (IVM) of mouse immature oocytes.

**Materials and Methods:**

In this experimental study, germinal vesicle (GV) oocytes from ovaries of a group of 25 female mice, 6-8 weeks of age, were dissected after being stimulated by 7.5 IU pregnant mare serum gonadotropin (PMSG) through an intraperitoneal (IP) injection. The fetal lung tissues were then prepared and cultured individually. A total number of 300 oocytes were cultured in the following three groups for 24 hours: control group (n=100) containing only base medium, group I (n=100) containing base medium co-cultured with 11.5- to 12.5-day old fetal mouse lung tissues, and group II (n=100) containing base medium co-cultured with 12.5- to 13.5-day old fetal mouse lung tissues. The proportion of GV and metaphase І (MI) oocytes matured into MІІ oocytes were compared among the three groups using analysis of variance (ANOVA). Correlation test were also used to evaluate the successful rate of IVM oocytes.

**Results:**

The proportions of GV oocytes reaching MІІ stage were 46, 65, and 56%, in control, I and II groups, respectively (P<0.05). The percentage of the oocytes remaining at the GV stage were higher in control group as compared with two treatment groups (P<0.05).

**Conclusion:**

This study indicated that fetal mouse lung tissue co-culture method increased the percentage of GV oocytes reaching MII stage.

## Introduction

Following the first successful clinical application of *in vitro* fertilization (IVF), Cha et al. (1) reported the first live birth from *in vitro* maturation (IVM) a decade later. In this advanced experimental technique, immature oocytes are extracted from the ovaries without need of gonadotropin stimulation. Various factors such as nutrients, energy substrates, vitamins, hormones and growth factors help *in vitro* development of oocytes (2,4). Fibroblast growth factor (FGF) family play an important role in setting the initial follicle growth, oocyte survival, proliferation and differentiation of granulosa cells, corpus luteum (CL) formation, steroidogenesis and angiogenesis (5,7). Many paracrine-acting factors have ability to arrange oocyte maturation and follicular development. More than 22 genes encode FGFs as significant paracrine regulators of angiogenesis, morphogenesis luteinizing hormone and reproduction (8,9). FGFs, especially FGF2 produced from granulosa cell, are involved in luteinizing hormone (LH) receptor expression, primordial follicle development and granulosa cell proliferation (10,13). FGF10 is a significant mediator of mesenchymal-epithelial signaling in various organs and tissues (14,15). In addition to the several members of the FGFs family, FGF receptors (FGFRs) have been implicated in mouse lung development (16). FGF10 is expressed at high levels from the earliest stages of lung development (15). Use of fibroblast co-culture and activin A promoted the growth and maintenance of preantral follicles in mice (17). Also, in a study by Heidari et al. (18) on evaluation of the effect of fibroblast co- culture system on the maturation and fertilization of oocytes from mouse preantral follicles, they have suggested that fibroblast co-culture increased the growth and survival rate of cultured preantral follicles in a significant manner by enhancement of granulosa cell proliferation. Retinoic acid is another factor playing a role on lung regeneration (19) and considered as an important modifier of vertebrate development, cell differentiation and tissue function (20). IVM with 9-cis-retinoic acid increased the developmental competence of the oocyte (21). Co-culturing is a method to support a long culturing period of IVM. *In vitro* co-culturing system has advantages in secreting trophic factors such as nutrients, substrates, growth factors, and cytokines as well as in eliminating toxins from the culture medium (22). We hypothesized that co-culturing with embryonic mouse lung tissue cells may promote immature follicle development due to presence of FGF and retinoic acid in early embryonic life of mouse life. The effect of embryonic lung tissue cells co-culture on IVM of immature follicles has not yet been studied in any species. Therefore, to improve culture conditions and develop appropriate culture systems with the idea of stimulating immature follicle growth, the present study aimed to investigate the effect of mouse embryonic lung tissue cells on *in vitro* growth of mouse immature follicles. 

## Materials and Methods

This is an experimental study that was approved by the Islamic Azad University, Science and Research Branch (Qaemshahr, Iran). All reagents were obtained from Gibco, UK, unless otherwise specified. 

Male and female NMRI mice were obtained from Pasteur’s Institute (Amol, Iran) and kept in the Central Animal House of Babol University of Medical Science (Babol, Iran) under a 12- hour light/12- hour dark regime at 20-24˚C, with adequate food ad libitum. 

### Culture medium condition

Base medium was consisted of alpha-Minimal Essential Medium (α-MEM, Gibco, UK), 100 μg/ml penicillin, 50 μg/ml streptomycin, and 4-(2-hydroxyethyl)-1-piperazineethanesulfonic acid (HEPES) supplemented with 10% fetal bovine serum (FBS, Gibco, UK). A total number of 300 oocytes were cultured in the following three groups for 24 hours: control group (n=100) containing only base medium, group I (n=100) containing base medium co-cultured with 11.5- to 12.5-day old fetal mouse lung tissues and group II (n=100) containing base medium co-cultured with 12.5- to 13.5-day old fetal mouse lung tissues. 

### Germinal vesicle follicle isolation

**Fig.1:**
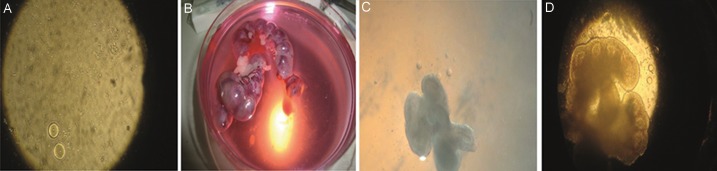
*In vitro* maturation (IVM) of immature oocytes and fetal mouse lung tissue co culture. A. Mouse embryos in the uterine horn oocytes at germinal vesicle (GV) stage, B. Immature oocytes before being co-cultured with the fetal mouse lung tissue, C. Immature oocytes with the fetal mouse lung tissue co-culture, and D. Oocytes at metaphase II (MII) stage.

Mice were stimulated by 7.5 IU pregnant mare serum gonadotropin (PMSG, Hipra, Spain) through an intraperitoneal (IP) injection. The animals were killed by cervical dislocation 48 hours later, while the ovaries were removed and placed on dissection medium consisting of α-MEM supplemented with 10% FBS, 100 μg/ml penicillin and 50 μg/ml streptomycin under mineral oil (Irvine Scientific, Belgium) to prevent evaporation, severe pH and temperature fluctuation. The germinal vesicle (GV)-stage oocytes were released by puncturing ovarian follicles with a 27G needle under a stereomicroscope and immediately transferred into 50 ml droplets of culture medium. A small pipette (slightly larger than the diameter of the egg) was applied to denude oocyte through rapid pipetting cumulus-oocyte complex (COC). After being washed 3 times, about 5 oocytes were placed in a 30 μl droplet for IVM (Fig .1A). 

### Isolation of fetal mouse lung tissue

According to previous study, the second phase (days 11-14 of gestational age) involves accumulation of retinoic acid from maternal circulation (23). Thus, 3 female and 1 male mice were placed in a cage for mating. Observation of vaginal plug was considered as day 0 of gestational day. After 10 to 14 days of gestation, the pregnant mice were killed. After dissection, the uterine horns under sterile conditions was removed (Fig .1B). Lung tissues was separated from embryos (Fig .1C). After washing, individual lung tissue sample along with 5 immature oocytes were placed in a drop of 30 μl under mineral oil and incubated at 37˚C in a humidified atmosphere of 5% CO_2_ in air.

### Assessment of oocyte maturation

Immature oocytes were classified as GV and GV breakdown (GVBD). Oocytes were cultured using inverted microscope (IX71, Olympus, Japan). Morphologic changes in the nucleus were evaluated with the release of the first polar body metaphase II (MII) as a measure of nuclear maturation of immature mouse oocytes. 

### Statistical analysis

The proportion of GV and GVBD oocytes reaching MІІ stage were compared among the three groups using analysis of variance (ANOVA). Correlation test were also used to evaluate the successful rate of IVM oocytes. All experiments were replicated 6 times. The statistical analysis was accomplished using the Statistical Package for the Social Sciences version 22 (SPSS, SPSS Inc., USA). A value of P<0.05 was considered statistically significant. 

## Results

As shown in Table 1, the proportion of GV oocytes (Fig .1C) reaching MІІ stage (Fig .1D) were 46, 65, and 56%, in control, I and II groups, respectively (P<0.05). The percentage of the oocytes remaining at the GV stage was higher in control group than the treatment groups (P<0.05). There is no significant difference in percentage of GVBD and degenerated oocytes among three groups (P>0.05). However, there were differences in oocytes remaining at GV stage among three groups, indicating that the oocytes remaining at the GV stage of control group were more than those of I (P<0.000) and II (P=0.002) groups. There was a significant different between the control and II groups regarding percentage of oocytes reaching MІІ stage, meaning that the number of MІІ oocytes in the group II were more than the control group (P=0.04). Correlation analysis showed that there is a reverse relation between GV and MІІ oocytes (R=-0.4), suggesting that increasing the number of MІІ oocytes decreased the number of GV oocytes (P<0.000). 

**Table 1:** Effect of fetal mouse lung tissue co-culture on *in vitro* maturation of mouse immature oocytes


Group	Cultured follicles	MII follicles	Degenerated follicles	GV follicles	GVBD follicles
	Number of immature follicles	n	n	n	n

Control	100	46^*^	4	29^*, **^	21
I	100	65^*^	2	11^**^	22
II	100	56	6	14^*^	24


GVBD; Germinal vesicle breakdown, MII; Metaphase II, *; P<0.05, and **; P<0.000.

## Discussion

It has been indicated that some growth factors play an important role in the final follicular growth. Several studies have shown the effect of some growth factors on IVM and development of immature oocytes (24,26). Our results demonstrated that the percentage of oocyte reaching MII stage were significantly higher in both experimental groups as compared to the control group. This finding support this principle that several members of the FGF family and FGFRs are involved in early mouse lung development (15). It has been also shown that FGFs and FGFRs are expressed in the developing lung, indicating that they are the major regulators of lung growth and differentiation (27). FGF10 as a specific group of chain- polypeptides that is bonded to heparin has an essential role in cell growth and tissue repair. FGF10 also stimulates ovarian granulosa cell differentiation (28) that leads to the expression of LH receptors and proliferation of ovarian germ cells (29). 

The establishment of an effective blood flow and formation of new blood vessels are necessary for ovarian tissue growth. Zeleznik et al. (30) have suggested that the selected follicles possess a more elaborate microvasculature than other follicles. In the studies by Yamashita et al. (31) and Schams et al. (32) they have indicated that pre- ovulatory follicle benefits from FGF2 and vascular endothelial growth factor (VEGF), so an increase in precursor hormones and nutrients lead to the mature follicle. Since a similar study that examined co-culture system with lung tissue was not found, and since the fetal lung like FGFs has growth factors effect on oocyte maturation, we focused on the articles on growth factors or other somatic cells present in co-culture system. Previous studies have shown the effects of basic fibroblast growth factor (bFGF) and retinoic acid on follicle growth *in vitro*. In addition, 5 µM of retinol showing antioxidant effects during bovine embryo culture improved blastocyst development (33). Modified 3D culture system during 8 days of *in vitro* culture of human follicles showed that growth, survival and viability of follicles were improved by bFGF (25). Zhang et al. (34) have showed that exposure to 50 ng/ ml FGF10 supplementation affected bovine oocyte maturation and increased the percentage of MII oocytes as compared to the control group. Adding 50 ng/ml FGF10 increased the proportion of oocytes with compressed chromatin after 6 hours. In a study by Nilsson et al. (13), a prepubertal 4-day-old rat ovary was cultured and treated with 40 ng/ml bFGF for 14 days and their results have showed varying degrees of follicle development, but the total number of follicles persection and the size of the ovary did not change. Their findings have also indicated that ovaries cultured with bFGF showed a significant decrease in the number of primordial follicles to 15 ± 2% that was followed by a corresponding increase to 85 ± 3% as compared to the control group. 

In another study, Matos et al. (35) have been shown the maintained morphological integrity of caprine preantral follicles after 5-day treatment with 50 ng/ml FGF2, stimulation of primordial follicles, and the growth of activated follicles. 

However, in a study by Nandi et al. (36), they have showed that oocyte maturation was affected by FGF alone as compared to the control, epidermal growth factor (EGF) and FGF+EGF groups. The application of the somatic cell co-culture system used in this study was also mentioned in some other studies. In a study by Duszewska et al. (22), they have demonstrated that the cleavage of bovine premature oocytes cultured with Vero cells were more than the control group, greater percentage of fertilized bovine oocytes developed into blastocyst, as well as greater percentage of bovine oocytes achieved inner cell mass layer. However, oocyte maturation *in vitro* is a dose-dependent growth factor and completely affected by culture conditions. Nandi et al. (36) have also reported that 20 ng/ml FGF increased the rates of nuclear maturation, cleavage and blastocyst formation in culture conditions of buffalo oocytes. At the 2 and 4 µM concentrations of all-trans retinoic acid, the rate of GV oocytes reaching MІІ stage were higher than the control group without any effect on the development of the fertilized oocyte on 2-cell stage (37). The purpose of choosing the fetal mouse lung tissue in this study was the presence of FGF and retinoic acid in early embryonic life. The maturation rates in the both treatment groups were more than the control group. Also, in group I, fetal mouse lung tissue co-culture showed a better response to oocyte maturation as compared to group II. It may be caused by the effect of FGF- induced secreted from fetal mouse lung in the early embryonic day. Accordingly, the stage of organogenesis occurred on days 10 to 14, while the creation of fetal mouse lungs starting from the day 9 was affected by FGFs secreted from lung tissue that continued to day 17. High percentage of MI and MII oocytes in experimental groups as compared to the control group may be attributed to fetal lung tissue due to the abundance of FGF secreted (38). 

## Conclusion

This study suggests that use of co-culture system with fetal mouse lung tissue increased the percentage of GV oocytes reaching MII stage. Comparison of the two treatment groups revealed that the group with the earliest stages of lung development had better response than the other treatment group. The evaluation of fetal mouse lung tissue co-culture system on IVM of mouse immature oocytes was verified for the first time. However, more research is necessary for the sanitation of IVM of immature follicles. 
